# The Provider Role and Perspective in the Denial of Family Planning Services to Women in Malawi: A Mixed-Methods Study

**DOI:** 10.3390/ijerph19053076

**Published:** 2022-03-05

**Authors:** Jill M. Peterson, Jaden Bendabenda, Alexander Mboma, Mario Chen, John Stanback, Geir Gunnlaugsson

**Affiliations:** 1 Department of Global Health and Population Research, FHI 360, Durham, NC 27701, USA; mchen@fhi360.org (M.C.); jstanback@yahoo.com (J.S.); 2 Faculty of Sociology, Anthropology and Folkloristics, University of Iceland, IS-102 Reykjavik, Iceland; geirgunnlaugsson@hi.is; 3 Department of Nutrition and Food Safety, World Health Organization, 1211 Geneva, Switzerland; jbendabenda@gmail.com; 4 Midwifery Department, Kamuzu University of Health Sciences, Lilongwe, Malawi; mbomaalexander1@gmail.com

**Keywords:** family planning, turnaway, sub-Saharan Africa, Malawi, provider, barrier

## Abstract

Family planning (FP) has been a global health priority for decades, yet barriers persist, including women being turned away from facilities without receiving services. This study assessed the provider role and perspective in client turnaway in three districts of Malawi. In 2019, data collectors surveyed 57 FP providers from 30 health facilities. All reported being comfortable providing FP to married women with children and married adolescents under 18 years old with children, whereas 12% of the providers expressed discomfort providing such services to married adolescents under 18 without children. Sixty percent of the providers required clients desiring FP and wishing to initiate oral contraceptives or injectables to be currently menstruating. Data collectors later conducted in-depth interviews (IDIs) with 8 of the 57 providers about client turnaway. During IDIs, providers’ most frequently mentioned reasons for turnaway was client pregnancy or suspicion of pregnancy. Providers expressed fears that initiating FP with a pregnant woman could cause community mistrust in the efficacy of modern contraception. Provider support for FP waned for nulliparous clients, regardless of age or marital status. To improve FP services in Malawi, providers need continuous education on all available methods of FP, a reduction in stockouts and programs to further sensitize the community to how contraception works. Understanding how Malawi has helped providers overcome social and cultural norms regarding provision of FP to adolescents might help other countries to make improvements.

## 1. Introduction

In 1994, the United Nations’ International Conference on Population and Development officially recognized family planning (FP) as a human right [1]. FP is widely acknowledged to be a key to success for several of the Sustainable Development Goals (SDGs), including those for ending poverty and hunger, promoting good health and achieving gender equality [2]. Yet, barriers to accessing FP, especially for women in low-income countries, have persisted.

The barriers to FP have been well documented and categorized, with slightly varying names or definitions, throughout the literature. Categories of barriers include those based on geography or distance from a facility; financial barriers due to the cost of traveling to the facility, missing work and/or the visit itself; misinformation, such as unfounded fear of side effects; medical barriers, including requirements for unnecessary procedures; and social barriers based on the role of women or adolescents in society [3,4,5].

Previous research outlines the many ways in which providers themselves can limit access to and choice of FP methods. For example, providers may require women to come with their husbands or the permission of their husbands to initiate a method, or they may require proof of menstruation (i.e., a soiled menstrual pad) at the time of the visit as a way to rule out pregnancy [3,6,7]. Providers may also insert their opinions on appropriate family size or timing of pregnancies into the discussion, including telling women it is time for them to have another child, or restrict the use of contraception for nulliparous women or women who have had fewer than the culturally expected number of children [3,8]. Marriage requirements and minimum age requirements have also been commonly imposed by providers [3,8,9,10]. Additionally, providers have been found to express bias in the method dispensed to the client, such as a preference for methods that are easier for the health worker to provide, such as injectables, compared to methods that require more effort by the provider, such as IUDs (intrauterine device), which necessitate a pelvic exam [11].

In Malawi, where the unmet need for FP was 18.7% in 2015–2016, and 53% of pregnancies were unintended, the government has prioritized increasing the uptake of FP and controlling rapid population growth [12,13,14]. In the early 1990s, the Malawian government implemented policy changes to liberalize the provision of FP services, which until that point were known as “child spacing” services [10]. Policy changes included removing restrictions on contraceptive services due to age or marital status, removing the requirement for a husband or guardian’s consent, and including an expectation that contraceptive services be provided daily and integrated into maternal and child health services. In the years following the policy changes, Tavrow et al. examined the issue of provider bias in Malawi [10]. Despite the policy changes, they found three-fifths of providers remained hesitant to provide FP to young, unmarried women without children, and two-thirds agreed “every method could be dangerous to someone” [10] (p. iv.). 

The government of Malawi made more recent policy changes related to FP, including encouraging the use of the “reasonably sure not pregnant checklist” in its “2010 Preservice Education Family Planning Reference Guide” [15]. In 2010, it allowed trained and paid community health workers (health surveillance assistants (HSAs)) to provide injectable contraception in a further attempt to make FP more accessible to all [16]. Additionally, the “National Sexual and Reproductive Health and Rights Policy 2017–2022” cites repositioning FP as a key development strategy and establishes a goal to strengthen “availability, access to, and utilization of family planning services at both facility and community level” [17] (p. 25). For Malawi to achieve its SDG targets, including to “ensure universal access to sexual and reproductive health and reproductive rights”, it will need to make certain that providers are not obstacles between women and their contraceptive preferences [18]. 

The purpose of this research study is to evaluate the role of providers in enabling FP method use and choice in Malawi, including describing provider experiences, attitudes and potential for bias. Specifically, we examine, from the providers’ perspective, the issue of turnaway—a client leaving a facility without a modern method of FP on the day she sought one. The term “turnaway” has been popularized in the context of access to abortion services in the United States, but here, we use it in the context of contraception [19]. Finally, we aim to describe providers’ discomfort in providing FP services to specific clients and why they feel it is sometimes necessary to turn women away without dispensing a contraceptive method.

## 2. Methodology

### 2.1. Design and Study Aim

This was a mixed-methods descriptive study that gathered data from FP providers at health facilities through a survey and in-depth interviews (IDIs) in three districts in Malawi. The data on providers were collected as part of a larger study that also collected data on FP clients to understand barriers leading to FP turnaway [5]. The aim of the research presented here was to better understand providers’ reasons for turning away women seeking FP, as well as their comfort level in providing FP to women with various characteristics (e.g., age, marital status and whether they have children). We also asked providers questions about the facilities where they work, as well as the services they provide, to better understand the realities of FP service implementation. Provider responses were written down verbatim, with minimal editing. When asking the providers about the facilities where they work, we were interested in what the providers themselves understood about the facilities, which may affect how they work, i.e., our unit of analysis in this study was the provider, not the facility. There was no attempt to validate providers’ responses with actual facility assessments. 

### 2.2. Sites

The study was conducted in 30 health facilities offering FP run by the Malawian Ministry of Health and Population—10 health facilities selected from each of the districts of Kasungu, Machinga and Zomba. These more highly populated districts were selected based on feasibility of data collection and diversity in characteristics of the districts, such as religion and modern contraceptive prevalence rates. The highest-volume FP sites in each district were identified by study staff based on recent annual aggregate-level District Health Information Software 2 (DHIS2) data of new FP clients and those restarting after a break of six months or more. Kasungu and Zomba districts were purposively selected for IDIs because they had higher turnaway rates than Machinga, while also accounting for logistical considerations [5].

### 2.3. Setting

Government policy states that FP services, methods and related materials are provided free to clients seeking care at public facilities in Malawi [13]. Initiating a new method of FP can be performed by clinicians (such as medical doctors), nurses or nurse-midwives (referred to here as nurses), pharmacists or pharmacy clerks, community-midwife assistants and HSAs. The method-specific training is the same for all, although nurses and clinicians receive training in general counseling to clients during their formal education that HSAs do not. Providers at both hospitals and health centers are supervised by managers in their respective facilities.

The traditional framework for provision of facility-based FP in Malawi begins with a group-counseling session. While not offered universally, facilities with enough client volume to warrant group counseling typically do so one or more days per week, after at least a few clients desiring FP have arrived at the facility. Group counseling normally covers a description of all methods, including how they work and what side effects one might expect with each. After hearing about all the methods, clients typically receive individual counseling in a private exam room, where a provider and client discuss the best method for the client and proceed with initiating the method, as appropriate. Given their roles in this process, providers are often the gatekeepers between women and contraceptive choice.

### 2.4. Study Sample and Data Collection

The study team aimed to survey two FP providers per site for a total of 60. Consistent with the sampling strategy, the sample size was intended to produce descriptive data and was not meant to achieve statistical generalizability. Upon arrival at the site on the day of survey data collection, data collectors asked the person in charge of the facility for the names of all FP providers, both present and not. Surveyed providers were selected from those present. If more than two FP providers were working the day of the visit, two were randomly selected using a Kish grid approach [20]. When only one provider was working the day of the visit, data collectors only surveyed one. 

We planned to conduct three to four IDIs per district in at least two districts, for a minimum of six to eight provider IDIs, to gain important insights on the provider’s role in FP provision to women. Within the selected districts, convenience sampling was used to select providers for IDIs, based on facility location and provider availability the day IDIs were conducted. On the day of IDIs, which took place approximately three to six months after the surveys, the data collector asked the person in charge for the first provider on the list who had been previously surveyed. If that person was not available for an IDI, they asked for the next person on the list. In all cases, they were able to speak with one of the two previously surveyed providers. 

Surveys and IDIs took place in a private space within the health facility or on the health facility grounds and were completed between October 2019 and April 2020. Surveys were conducted on electronic tablets using Open Data Kit (ODK) [21]. IDIs were recorded and transcribed by the data collectors. Providers participating in IDIs were compensated MWK 7000, or approximately USD 10, for their time, as required by the Malawian Institutional Review Board, National Health Sciences Research Committee.

### 2.5. Analysis

Survey results were downloaded as .csv files. Open-ended re-coding was conducted in Excel by two researchers prior to results being uploaded into SPSS, Version 26.0, where descriptive analyses were conducted [22]. Given that sampling was based on those present at the time of data collection, we assessed whether some types of providers (e.g., clinicians, midwives, HSAs or nurses) were more likely to be present and available for sampling and data collection than others to determine the representativeness of the FP provider population. IDI transcriptions were coded by one researcher with the use of NVivo version 12 [23]. The researcher performing the coding consulted a second researcher when the responses were not clear or further interpretation was warranted. Researchers used a grounded theory approach, whereby general themes and frequency of responses were analyzed based on the coded responses to the questions. Researchers assessed the data for thematic saturation using the method outlined by Guest et al., with a threshold of ≤5% new information to indicate saturation [24]. 

## 3. Results

### 3.1. Provider Survey

We conducted quantitative surveys with 57 FP providers; three facilities had only one FP provider present the day of the surveys. We did not find notable differences (data not shown) in the type of providers present or absent the day of the survey. We were able to capture a range of provider cadres providing FP. In the selected facilities, most providers (84%) were working in health centers. All but three had been providing FP services for at least one year, and among those, the average number of years providing was 5.78, but the range extended to 24 years. The majority (53%) were nurses, with another quarter self-reporting as HSAs (Table 1).

Providers were nearly evenly divided in the frequency with which they reported availability of FP services (Table 1). Different providers working for the same facility answered this question differently at 11 of the facilities. When asked about the number of clients attending FP group counseling sessions, 78% of providers reported averaging more than 15 clients per session. On facility-designated FP days, 90% of providers reported personally seeing more than 10 clients. Fewer than half of providers (47%) reported that group counseling was offered daily at their facility. The mean number of FP providers available on FP days to see clients was 5.8 (standard deviation = 7.0, range 1 to 36). 

The methods most reported by providers as normally available at the facilities were condoms, oral contraceptive pills (OCPs) (combined), injectables (intramuscular, IM) and implants. Of those methods, the most common stockouts were of injectable (IM) and Jadelle implants (Table 2). Almost half of providers (47%) reported at least one day in the past week when pregnancy tests were not available, affecting 20 of the 30 facilities. When asked if providers charge clients for a pregnancy test, 9% of providers said yes, and an additional 5% reported asking clients to buy one when they run out at the facility. The providers who did so reported charging between MWK 400 and MWK 1500 for the pregnancy tests (approximately USD 0.50 to USD 2). 

When providers were shown a copy of the “reasonably sure not pregnant” checklist included in the pre-service training manual and asked if they had seen it before, 28% reported they had. Of those, 94% felt comfortable using it, and 87% could explain to the data collector how to use it. 

#### 3.1.1. Requirements for Clients Initiating Various Methods

Data collectors asked providers in an unprompted manner what requirements they had for clients initiating OCPs, injectables, implants and IUDs. The results in Table 3 are for providers reporting their facility regularly offered the method. 

Some sort of medical eligibility was mentioned by 67% to 95% of providers for the four methods. Examples of medical eligibility include no history of blood clots, no current sexually transmitted infection (STIs) and no problems with headaches or migraines. Negative pregnancy tests were mentioned as a requirement more often than current menstruation, the “reasonably sure not pregnant checklist” or a negative pregnancy test if not currently menstruating, as means to ensure a woman was not pregnant. Nearly half (47%) of providers, however, reported a stockout of pregnancy tests on at least one day in the past week. 

#### 3.1.2. Reasons Providers Were Uncomfortable Providing Certain Clients with Services

**Figure 1:**
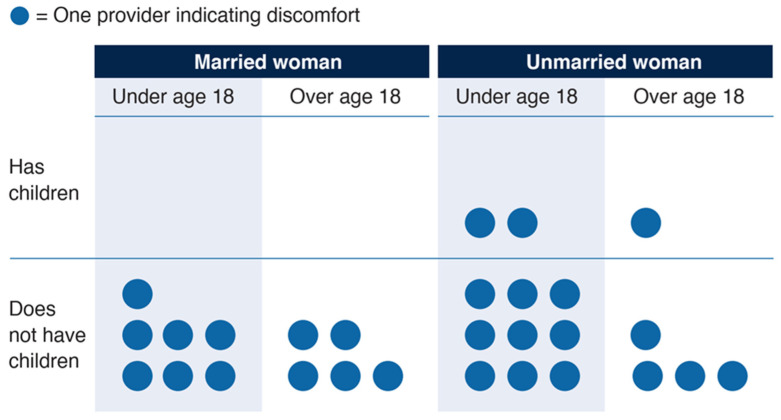
Providers who responded they were uncomfortable providing FP services to each type of client.

None of the 57 providers expressed being uncomfortable providing FP to married women with children nor married adolescents under 18 with children (Figure 1). Conversely, seven (12%) providers expressed discomfort providing FP to married adolescents under 18 without children. The reason most often given was “she should have children,” with three providers citing this reason. Providers were most likely to express discomfort providing FP to unmarried adolescents under 18 with no children, with nine (16%) providers expressing being uncomfortable. The reason cited by seven providers was “she should not be having sex”. Three felt uncomfortable because “it may be bad for her health”. When considering unmarried adolescents under 18 with children, however, only two providers expressed discomfort, with one citing “she should not be having sex” and one citing “it may be bad for her health”.

As indicated by the “uncomfortable” responses displayed in Figure 1, providers expressing discomfort were most often uncomfortable with clients who did not have children. No cadre of provider expressed discomfort more frequently than the others. HSAs, nurses and other providers all experienced discomfort.

### 3.2. Provider In-Depth Interviews

In-depth interviews were conducted with eight FP providers—four in Kasungu and four in Zomba. As described by Guest et al., using a base size four (analyzing four IDIs) and run length of two (adding results from two more IDIs to determine how much new information was added) as evaluation parameters, we reached the ≤5% new information threshold (3%) at six interviews, providing confidence that our sample reached thematic saturation [24]. 

Providers’ years of experience in providing FP ranged from 1 year (although she had previously worked in obstetrics for 25 years) to 9 years. Five were nurses, one a clinician and two FP providers; six were female and two were male. While some providers had received training on FP while in school, more recent experiences varied between formal refresher training and on-the-job training. The methods respondents reported as providing corresponded with the methods on which they had been trained. The method-specific training mentioned, including that for implant insertion and removal, and subcutaneous injectables, had been sponsored by nongovernmental organizations.

The thematic analysis identified four domains most discussed by providers: the client types some are uncomfortable providing contraception for, reasons providers turn clients away without a method, community-held beliefs and myths about FP, and areas for service improvement.

#### 3.2.1. Client Types Providers Are Uncomfortable Initiating on Family Planning 

The interviewers followed up on the survey results of providers uncomfortable with providing FP to certain types of clients, including those who were unmarried, without children or adolescents. All eight providers expressed that medically eligible women should be treated equally and given a method. One noted, “the golden rule is everyone who wants to have a FP method should get it”. Another said, “Yes, because they have a right…I cannot deny them”. 

Despite this, some providers continued to express unease with providing a method to adolescents. For example, one provider expressed feeling particularly uneasy when providing FP to young adolescents (13–14 years old), because of what being sexually active might mean for the girls’ ability to do well in school.

*As for me, teenagers are very hard for me to provide the method most of the time. I still feel they may not concentrate on school or what they’re doing. Some of them are very young like 14; some of them are as young as 13. So it’s very hard for me to provide the methods to them though I still provide. Most of the health workers there, we even ask each other, should I still provide? None of us are comfortable providing to teenagers because we just become afraid, I don’t know why. Sometimes we are afraid that maybe they might have uterine fibroids, and sometimes we are afraid that they may not continue school and not concentrate.*

The provider went on to say,

*Personally, it’s not been medically proven but most of us just worry that they might have problems when they get married and want a baby. Fertility might not return as quick as we think, or most of us are afraid that maybe because they protect themselves against pregnancy, but are they protecting themselves against the STIs?*

Another provider expressed discomfort with indecisive clients,

*Sometimes I am uncomfortable to provide permanent methods to the indecisive ladies. If she says I haven’t really talked to my husband, then I tell her to go back so she can discuss it with her husband. *

#### 3.2.2. Reasons for Turnaway

The most frequently mentioned reason for turning away clients seeking contraception, mentioned by seven out of eight interviewed providers, was when the woman tested positive for pregnancy or showed signs of pregnancy. Two providers mentioned turning away women who had missed their last period, and two mentioned turning away those who were not currently menstruating. According to one provider, some women seek FP due to a belief that initiating contraception while pregnant will induce an abortion. As a result, providers reported insisting on pregnancy tests for anyone with a missed period before dispensing a method.

*Because of myths we find a lot of women who are already maybe one month pregnant, but most think that if we give them a method they will abort. That’s why after one or two months you see a good number of women who come wanting to be put on Implanon, but after assessing you find that they are pregnant. And they are open, they tell you that I thought that if you put Implanon in me the pregnancy will go away.*

*…for some, they would say they are suspecting themselves to be pregnant so we offer them [a pregnancy test] to rule out pregnancy, and sometimes when they are saying that they are having menses we would even see, because some can cheat to say that they are having menses just to get a method, which is not good for them. *

Additionally, women who are already pregnant and initiate a method may conclude that FP does not work.

*And mainly they come back to say that your methods are not working, that’s why I’m pregnant, so we have to make sure that sometimes they’re not pregnant and then we give a method.*

Six of the eight providers accurately listed clinical reasons for turning away clients, such as elevated blood pressure when seeking hormonal methods, or sexually transmitted infections when seeking an IUD. One incorrectly reported that FP is less effective with older women due to their “weak uteruses”. 

Six providers mentioned stockouts as a reason for turnaways, most frequently citing injectables (cited by six providers), but IUDs (cited by four providers) and the materials needed for IUD insertion (cited by two providers) were also mentioned as having been recently stocked out. 

One provider discussed turning away clients whose husbands were not aware of their wives’ use. The explanation given was,

*…especially implants the women cannot hide it because someone can see it, so if the husband realizes that the wife has a family planning method it brings conflicts to the family, which will force the woman to come seek for removal when the days are not due.*

Arriving late, not having a health book and no availability of a trained provider for a particular method were each mentioned by one of the eight providers as reasons for turnaway. 

Based on the survey results, we followed up in the IDIs on the possibility of being turned away for refusing HIV testing. Providers reported that knowing one’s HIV status was recommended when initiating FP, but refusing a test or not knowing her status would not be a reason to deny someone a method. Instead, the information is used to help women choose the most effective method if taking antiretrovirals. 

#### 3.2.3. Provider Reports of Community-Held Beliefs and Myths about Family Planning

Providers were asked about beliefs and myths shared in the community regarding FP. In total, 13 beliefs or myths were identified about FP. Four providers mentioned the belief that only women who have had children, or a certain number of children, should use FP; one noted a belief that women should have four or five children before they get a bilateral tubal ligation (BTL). Another provider noted that use of youth-friendly services by nulliparous adolescents provides evidence that this belief may be changing in communities. Two providers discussed a community-held belief that using FP will reduce a woman’s sex drive. Box 1 contains beliefs mentioned by individual providers.

Box 1 **Community-held beliefs and myths about FP, as explained by FP providers**

- Only women with children (or a certain number) should use FP.
- Using FP reduces a woman’s sex drive.
- Young people should not use FP; they might be ridiculed and called prostitutes if they are known to be using FP.
- Initiating a method can induce an abortion.
- Implants and IUDs can cause pain to men during intercourse.
- FP causes the uterus to swell.
- Implants can migrate to the heart or stomach.
- FP methods used by women can cause impotency in men.
- OCPs can accumulate in the stomach.
- In the community, there is a preference for methods provided by the witch doctor.
- If a woman initiates soon after delivering a child, she might be promiscuous as local custom says sexual relations should not re-start for 6 months.
- FP can cause sterility.

#### 3.2.4. Areas for Service Improvement 

When asked what they would need to improve in the services they offered, providers most frequently mentioned training, reducing their workload and reducing stockouts of methods and supplies. Some facilities do not have anyone available to insert IUDs or perform BTL, and some are overly dependent on one or two providers who are trained on these methods. For example, one provider expressed frustration that when the sole provider trained to provide BTL at their facility had been transferred to another facility, his/her facility was no longer able to provide that method. In addition, one provider expressed a desire for more exam rooms, as inserting long-term methods can take a while, leaving other clients to wait for extended periods. 

Another provider highlighted the importance of improving community education on FP, noting community members were unaware of how the methods worked and that they would not work if a woman was already pregnant. A different provider lamented the myths that spread about particular methods and how that affects the service volume faced by providers.

*The misconceptions and the myths, they are still circulating in the villages. If somebody sees a side effect of a certain family planning method, she may choose to discourage friends who want to take the same method. You may see a lot of women who have been told by their friends about side effects hence discouraging them to take the method which are we are encouraging women to take. One example is Jadelle. One year you will see a woman has come who has taken Jadelle, and next year she is coming and saying it is doing me such bad things in my body. When you ask, you see that she has just gotten the message from a friend it will kill you, so that’s our major challenge. We may waste a lot of Jadelles because of these myths and misconceptions.*

Given the pressure in the community to restrict adolescent FP use, one provider reported coming in extra hours to see adolescents at times when their confidentiality could best be protected.

## 4. Discussion

This mixed-methods study explored providers’ level of discomfort in initiating FP methods with various categories of clients and other reasons for client turnaway. We found providers to experience the most discomfort when initiating contraception with nulliparous women, with little to no discomfort when initiating contraception with other categories of women based on marital status or age. Providers reported that service delivery would improve with reduced workload and frequent stockouts of at least one method of FP. Both factors plausibly contribute to client turnaway. This finding is supported by our research on client turnaway, showing clients were most often turned away due to a lack of providers or methods and rarely for reasons such as age or parity [5]. The most frequently mentioned reason for turnaway by providers during IDIs was client pregnancy or suspicion of one. 

Nearly 30 years have passed since research by Tavrow et al. described provider actions as discouraging, delaying or denying women FP [10,25]. Hazel et al. found continued evidence of provider mistreatment of clients in their research in six districts of Malawi in 2018, including refusal of service, not accounting for the client’s method preference and humiliation, such as verbal abuse, by the provider [26]. Our research more closely followed the issues noted in the Tavrow study and found improvement on those issues, especially in their openness to provide FP methods to adolescents. None of the providers we surveyed reported requiring parental consent before dispensing FP to adolescent girls, whereas more than a quarter believed they needed it in the mid-1990s [10]. Less than a fifth of providers expressed concerns about providing FP to unmarried adolescents under the age of 18 without children—down from 40% of providers who had expressed discomfort with this group in the Tavrow et al. research [10]. Although the methodologies of the studies were different, all of the providers we spoke with during IDIs noted the obligation of providers to give FP to all women who wanted it and who were clinically eligible.

According to providers, however, this openness to FP is not equally supported in the community and notably not for adolescents. Adolescent pregnancy is a problem in Malawi, where 29% of women aged 15–19 years have begun childbearing [12]. Research in one district of Malawi on the subject of adolescent reproductive and sexual health summarized the conflicting cultural viewpoints that place a high value on both motherhood and education but, by law, mandate a 12-month absence from school when girls become pregnant [27]. Similarly, sexual relations for adolescents are discouraged, but sexual initiation ceremonies for girls are commonly practiced around the time of first menses, and transactional sex with older men is common [27]. These complexities are reflected in the responses of the providers we interviewed who worried whether an adolescent girl in need of FP would be able to concentrate on schooling. 

As the survey indicated, the factor that most caused providers unease was dispensing FP to nulliparous clients, regardless of other factors, such as marital status or age (Figure 1). Motherhood is highly valued in Malawi, and there are superstitions around not having any children in a marriage; having several children is the ideal for most [28,29]. While the mean ideal number of children per family has declined from 5.1 in 1992, it remained at 3.7 in 2015–2016 [12]. Social and cultural norms are known contributors to provider bias, but as demonstrated in research conducted in Ghana in the late 1990s, a lack of understanding of the science behind how contraception works, or an inadequate belief in the science, can also lead to provider bias [9,11,30]. Beliefs persist in Malawi that initiating modern FP methods too young may lead to infertility, or that one should “know” their fertility by having a child before starting to use FP [27,29]. Providers did not mention parity in their reasons for turning away clients in the quantitative survey, but during the IDIs, half of the providers cited a belief that women should have children, or a certain number of children, before using FP. Although one provider acknowledged this is not supported by science, this belief likely rests on the fear that young girls could become infertile in the future because of early FP use. 

During IDIs, providers articulated beliefs and myths held by community members about FP. Although several of the myths were mentioned by only one provider, they are the same or similar to FP myths noted in the literature [31,32,33]. In addition, providers noted that if community members had a better understanding of FP and how it works, they would not need to spend so much time inserting and removing implants, for example, due to rumors women hear in the community about the method. Further, they noted that community members might accuse a young girl in need of FP of being a prostitute and said that community members often did not understand how various methods worked, instead believing misguided myths concerning FP. 

Establishing that a client was not pregnant was also important to providers. When available, providers mentioned requiring negative pregnancy tests. They also relied heavily on proof of menstruation to rule out pregnancy. That 14% of providers reported either charging for pregnancy tests or asking clients to provide their own, when they should be free and available, is a potential barrier to accessing FP. Some responses to the question varied among providers working at the same facilities, indicating that charging for pregnancy tests may not be a facility-level practice, but rather implemented by individual providers. Research in Malawi based on the value of the dollar in 2016 showed the procurement cost of pregnancy tests in Malawi ranged between USD 0.08 and USD 0.25—less than the USD 0.50 to USD 2 providers in our survey reported charging [34]. 

The qualitative results, however, provide a rationale for the importance of ensuring a client is not pregnant before she initiates a method—the providers’ concern that if they initiate a pregnant client on FP, she will tell others in the community that modern FP methods are not effective. Providers claimed this can cause the community to lose trust in the efficacy of modern contraception. In addition, providers reported instances of women seeking a method of FP in hopes of inducing an abortion despite neither OCPs, injectables nor implants being abortifacients. Another consequence of pregnant women seeking FP with hopes of inducing an abortion is that they may delay seeking antenatal care in the meantime. 

Few providers, however, reported having heard of another method of ruling out pregnancy—the “reasonably sure not pregnant” checklist—despite it being included and frequently mentioned in their pre-service training materials [15]. Better familiarity with, and use of, the checklist could help providers bridge the gap when pregnancy tests are not in stock or in cases when pregnancy can easily be ruled out without the use of a hormonal test. A refresher training on the checklist, along with support for its use from provider champions, as suggested by Carlough and Jacobstein, could be particularly impactful in allowing women without risk of pregnancy to initiate modern FP [30]. 

Other provider requirements for initiating a method, beyond medical eligibility, were few. Although about a third of providers mentioned requiring an HIV test, offering one is part of the regular FP initiating protocol. When this matter was explored further in the IDIs, providers reported they would not deny a method to a client who had not had a recent HIV test, but rather they used information on HIV status to recommend an appropriate method for those on antiretrovirals. This is again supported by client surveys on reasons for turnaway, which showed no women citing the primary reason for turnaway being refusal of an HIV test [5].

In several cases, providers working at the same facilities answered questions about that facility differently, including the frequency with which FP services are available and the availability and stockout of methods and pregnancy tests. This emphasizes the unevenness in which these services may be delivered by individual providers. Method stock may fluctuate during the week; pregnancy tests may be accessible to some providers and not to others within the same facility; and only some providers may be trained to provide particular methods. Providers also reported daily offer of group counseling more than daily offer of FP services, which may reflect its inclusion in routine health education talks, antenatal care services and routine growth monitoring.

Our results showed Malawi doing better in some ways suggested by Carlough and Jacobstein to address provider bias than in others [30]. Providers expressed a desire for more training on methods they are not familiar with, which, coupled with championing providers and a stronger focus on the benefits of FP in relation to risks, would undoubtedly result in better service and fewer turned-away clients. A more regular supply of commodities would help providers in their same-day service provision, as would facilities to accommodate more clients simultaneously, rather than a single family-planning exam room. 

This study benefited from a mixed-methods approach and is the first study to re-examine the reasons for client turnaway in Malawi studied by Tavrow et al. (1999). It is a strength that it explores client turnaway, and specifically, the proportion of clients turned away for particular reasons, information that has been lacking in the literature. Similarly, barriers to family planning have not been frequently examined since the 1990s and early 2000s. Another strength of this research is that it was conducted in 30 facilities, stretching across three districts. Although we acknowledge our study has limited statistical generalizability beyond the three districts and clients served in other types of facilities, the selected facilities are typical of the types found across the country. Alternative methods for collecting data on provider requirements for initiating various contraceptive methods, such as simulated clients or direct observation, were not possible; these might provide more reliable data but come with their own drawbacks. IRB approval for simulated clients has proven difficult, despite it being employed in quality assessments in Malawi. Direct observation can be uncomfortable for clients and introduces the risk of the Hawthorne effect, whereby a person being observed may modify their behavior as a result of being observed [35]. 

## 5. Conclusions

Providers were generally supportive of FP for anyone desiring and medically eligible to use it, but their support waned for nulliparous clients, regardless of age or marital status. Understanding how Malawi is able to help providers overcome social and cultural norms regarding the provision of FP to adolescents, in particular, would help other countries to determine whether similar interventions might prove successful in their own settings. Provider concerns in dispensing FP to nulliparous clients focused on fears of future sterility and mistreatment in the community if young women were discovered to be using contraceptives. Further education and sensitization of the community to how contraceptive methods work, as well as dispelling common myths and encouraging more acceptance of the use of FP, may also put providers’ minds at ease and allow them to feel less out of step with their communities. Providers expressed a desire for more trained providers in all methods, a reduction in stockouts of methods and supplies, and more exam rooms to accommodate the full client load. Investigation should also be conducted into how providers can be better supported. 

## Figures and Tables

**Table 1:** Surveyed FP provider service statistics by district in Malawi, October to December 2019.

Variable	Total	Zomba	Machinga	Kasungu
	N = 57 *n* (%)	N = 19 *n* (%)	N = 20 *n* (%)	N = 18 *n* (%)
**Responses**	57 (100)	19 (33)	20 (35)	18 (32)
Facility Type				
Hospital *	3 (5)	0 (0)	2 (10)	1 (6)
Health center	48 (84)	15 (79)	16 (80)	17 (94)
Health post	2 (4)	0 (0)	2 (10)	0 (0)
Dispensary	4 (7)	4 (21)	0 (0)	0 (0)
What is your professional cadre?				
Nurse	30 (53)	13 (68)	10 (50)	7 (39)
HSA	14 (25)	2 (11)	8 (40)	4 (22)
Clinician	9 (16)	3 (16)	2 (10)	4 (22)
Community midwife assistant	3 (5)	1 (5)	0 (0)	2 (11)
Pharmacy clerk	1 (2)	0 (0)	0 (0)	1 (6)
How often are family planning services available at this facility?				
Daily	21 (37)	11 (58)	8 (40)	2 (11)
Weekly	17 (30)	2 (11)	7 (35)	8 (44)
More than once a week but not daily	19 (33)	6 (32)	5 (25)	8 (44)
On the days you provide family planning services, how many clients do you personally see on average?				
1 to 5	1 (2)	0 (0)	0 (0)	1 (6)
6 to 10	5 (9)	1 (5)	1 (5)	3 (17)
More than 10	51 (89)	18 (95)	19 (95)	14 (78)
How often is family planning group counseling offered?				
Group counseling not regularly offered	6 (11)	4 (21)	1 (5)	1 (6)
Daily	27 (47)	11 (58)	8 (40)	8 (44)
Weekly	11 (19)	2 (11)	4 (20)	5 (28)
More than once a week but not daily	13 (23)	2 (11)	7 (35)	4 (22)
On average, how many women attended each group counseling session?				
	N = 51	N = 15	N = 19	N = 17
10 or fewer	0 (0)	0 (0)	0 (0)	0 (0)
11 to 15	11 (22)	8 (53)	2 (11)	1 (6)
More than 15	40 (78)	7 (47)	17 (90)	16 (94)

* Hospital refers to district or rural hospitals. Central hospitals were not included.

**Table 2:** Provider (N = 57) report of methods normally available and stocked out in Malawi, October to December 2019.

Method	Method Normally Available at this Facility? N = 57	Has a Method Normally Available Been Stocked out in the Past Week?
	*n* (%)	*n* (%)
Condoms	57 (100)	0 (0)
OCP *-combined	57 (100)	2 (4)
Injectable-IM	54 (95)	12 (23)
Implant-Jadelle	53 (93)	14 (27)
Implant-Implanon	53 (93)	5 (9)
OCP-Progesterone only	51 (89)	5 (10)
Emergency contraception	34 (60)	4 (12)
Injectable subcutaneous	26 (46)	1 (4)
IUD *	19 (33)	1 (5)
Tubal ligation	11 (19)	2 (18)
Vasectomy	6 (11)	2 (33)

* OCP = Oral contraceptive pills; IUD = Intrauterine device.

**Table 3:** Provider requirements for FP initiation at facilities that regularly offered the method, by method, in Malawi, October to December 2019.

	OCP *	Injectable	Implant	IUD *
	N = 57	N = 57	N = 53	N = 19
What requirements do you have for women initiating the method specified?				
	*n* (%)	*n* (%)	*n* (%)	*n* (%)
Medically eligible	38 (67)	40 (70)	42 (87)	18 (95)
Negative pregnancy test	39 (68)	38 (67)	36 (68)	13 (68)
Currently menstruating	34 (60)	34 (60)	24 (45)	9 (47)
Reasonably sure not pregnant	18 (32)	20 (35)	14 (26)	4 (21)
Negative pregnancy test if not currently menstruating	11 (19)	14 (25)	13 (25)	4 (21)
HIV test	12 (21)	16 (28)	20 (38)	7 (36)
Pelvic exam	6 (11)	5 (9)	5 (9)	6 (32)
Already has children	1 (2)	1 (2)	2 (4)	1 (5)
Spousal consent	1 (2)	1 (2)	1 (2)	0 (0)
Parental consent if under 18	0 (0)	0 (0)	0 (0)	0 (0)
Immunization	0 (0)	0 (0)	0 (0)	0 (0)
None	0 (0)	0 (0)	1 (2)	0 (0)

* OCP = Oral contraceptive pills; IUD = Intrauterine device.

## Data Availability

The data presented in this study are openly available in Harvard Dataverse [doi:10.7910/DVN/S7NXKL].
